# Intersektoralität im Fokus – Strategien und aktuelle Forschungsprojekte zur Versorgung geriatrischer Personen

**DOI:** 10.1007/s00103-024-03851-3

**Published:** 2024-03-13

**Authors:** Jenny Unterkofler, Miriam Hertwig, Leo Cornelius Bollheimer, Jörg Christian Brokmann

**Affiliations:** 1 Klinik für Anästhesiologie, Uniklinik RWTH Aachen (NRW), Aachen, Deutschland; 2 Zentrum für klinische Akut- und Notfallmedizin, Uniklinik RWTH Aachen (NRW), Aachen, Deutschland; 3 Klinik für Altersmedizin, Uniklinik RWTH Aachen (NRW), Aachen, Deutschland

**Keywords:** Demographie, Intersektorale Versorgung, Hochaltrige, Telemedizin, Versorgungsnetzwerk, Demographics, Intersectoral care, Oldest-Olds, Telemedicine, Care network

## Abstract

In Anbetracht des demografischen Wandels wurde bereits die Notwendigkeit zur intersektoralen Versorgung der alternden Bevölkerung identifiziert. Die Strategien zur Umsetzung sind vielfältig und setzen an verschiedenen Hebeln an, die jeweils unterschiedliche Sektorenüberschneidungen voraussetzen. Der Artikel bietet einen Überblick über bereits abgeschlossene und noch laufende Projekte zur Versorgung geriatrischer Patienten. Dabei wird deutlich: Der Aufbau von Netzwerken als unverzichtbare Basis für Intersektoralität kann nicht in direkten Interventionseffekten messbar gemacht werden und erschwert somit den Kosten-Nutzen-Nachweis. Ebenso zeigt sich, dass einige Forschungsprojekte bei der Überführung in die Regelversorgung durch finanzielle und personelle Engpässe scheitern.

Brauchen wir in Deutschland ein Umdenken oder weniger innovationsbezogene Förderlinien zur besseren Implementierung und Erforschung bereits existierender Konzepte? Internationale Vorbilder wie Japan machen klar: Die Kostenreduktion bei der Versorgung der alternden Bevölkerung sollte langfristig betrachtet werden und bedarf kurzfristig erhöhter Finanzvolumina. Für eine nachhaltige Implementierung von sektorenübergreifenden Ansätzen im Alltag sollte deshalb die Versorgungsforschung fest(gefahren)e Strukturen, Abläufe und Finanzierungen neu ordnen. Durch Verknüpfung der unzähligen Projekte und Ideen verschiedener Bereiche könnte in Zukunft eine Realisierung des Anspruchs der intersektoralen Versorgung geriatrischer Patienten erreicht werden.

## Abstract (en)

In view of the demographic change, the need for intersectoral care of the aging population has already been identified. The strategies for implementation are diverse and address different approaches, each of which requires different sectors to overlap. This article provides an overview of already completed and ongoing projects for the care of geriatric patients. It becomes apparent that the development of networks as an indispensable basis for intersectoral care cannot be measured in terms of direct intervention effects and therefore makes it difficult to prove the cost-benefit. It is also evident that some research projects fail to be implemented into standard care due to financial and staff shortages.

Do we need a rethinking in Germany or less innovation-related funding lines for better implementation and research of existing concepts? International role models such as Japan show that cost reduction for the care of the aging population should be considered in the long term, which requires increased financial volumes in the short term. For a sustainable implementation of cross-sectoral approaches into everyday life, research should therefore reorganize tight and/or entrenched structures, processes, and financing. By linking the countless existing projects and integrating ideas from different sectors, future demands of intersectoral geriatric care may be achieved.

## Einleitung

Der demografische Wandel stellt global neue Anforderungen an die Medizin. Anstrengungen zur besseren intersektoralen Versorgung alter Menschen sind daher anzustreben. In Deutschland gibt es viele Angebote für die sogenannten Oldest-olds (Alter ≥ 80 Jahre [1]). Diese werden jedoch häufig von Leistungserbringern aus verschiedenen Sektoren unabhängig und überschneidend angeboten, die (digitale) Vernetzung bleibt schwer überschaubar. Im Rahmen der Digitalisierung in der Medizin soll sich dies nun ändern – Telemedizin, digitale Dokumentation und Rezeptierung sind nur ein Teil davon. Insbesondere die intensivierte Verzahnung bereits existierender Strukturen ist essenziell für eine kosteneffektive und verbesserte Versorgung. Dies ist Gegenstand der aktuellen Empfehlungen des Sachverständigenrates zur Begutachtung im Gesundheitswesen sowie der vierten Stellungnahme der Regierungskommission, die eine intersektorale Neuregelung der Akut- und Notfallversorgung durch die Etablierung integrierter Notfallzentren und Leitstellen adressiert [2, 3]. Doch trotz aller Forderungen und finanzieller Unterstützung von Forschungsprojekten in diesem Bereich erscheint das Ziel der intersektoralen Versorgung für geriatrische Personen noch nicht erreicht. Dieser Artikel soll einen Überblick über das breite Spektrum aktueller nationaler Lösungsvorschläge für die optimierte intersektorale Versorgung geriatrischer Personen geben und analysieren, woran die flächendeckende Umsetzung derzeit noch scheitert.

## Intersektoralität

**Figure 1:**
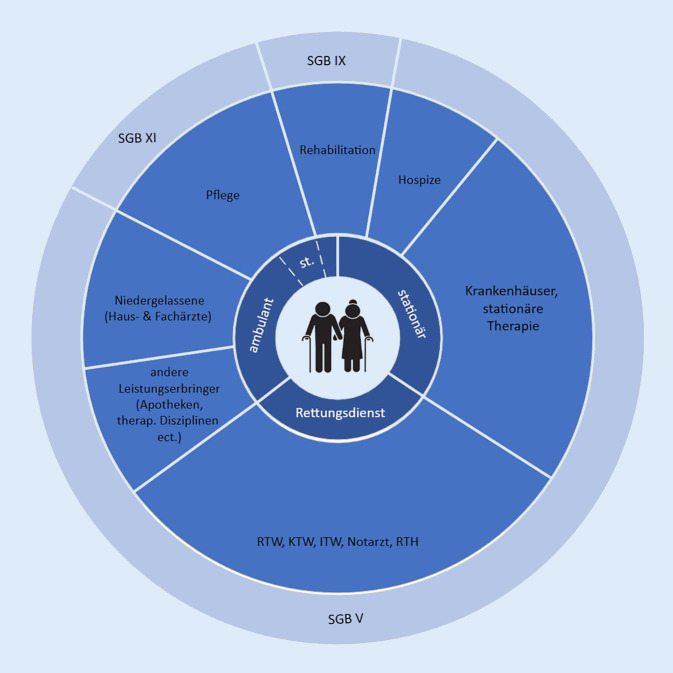

Die Problematik beginnt bereits bei der nicht allgemeingültigen Definition von „Intersektoralität“, denn die Grenzen verschwimmen nicht nur zwischen den Akteuren, sondern auch zwischen den juristischen Verankerungen der verschiedenen Sozialgesetzbücher (SGB I bis XII (respektive XIV)). So werden sämtliche Leistungen der medizinischen Versorgung (stationär, ambulant, Krankenfahrten, Physio- und Ergotherapie etc.) im SGB V geregelt, alle Pflegeleistungen (ohne Hospize) jedoch im SGB XI und die Rehabilitation in SGB IX [4]. Der Austausch zwischen den einzelnen Leistungserbringern betrifft alle Dimensionen der Versorgung, ist häufig zeitversetzt und fokussiert meist nur einen Teilaspekt der gesundheitlichen Situation [5]. Technische Herausforderungen an den Schnittstellen, fehlende Interoperabilität und Datenschutzvorgaben sind nur einige Hürden, die es zu überwinden gälte. In der letzten Legislaturperiode befasste sich eine Bund-Länder-Arbeitsgruppe mit der Thematik und erarbeitete Vorschläge zur Weiterentwicklung der telematischen Infrastruktur [6]. Ein erstes Eckpunktepapier wurde 2019 konsentiert, konkrete Umsetzungen gibt es derzeit nicht [7]. Die intrinsische Trennung der Sektoren führt häufig zu Versorgungsbrüchen am Sektorenübergang, was insbesondere bei Patienten mit komplexen chronischen Krankheitsbildern und Pflegebedürftigkeit sichtbar wird (Abb. 1; [8]).

Die Sicherstellung der gesundheitlichen Versorgung der Bevölkerung im bisher typischen Umfang kann nur durch einen sektorenintegrierenden Ansatz gelingen. In dessen Mittelpunkt muss der Bedarf der Patienten stehen und ein kontinuierlicher, ressourcenschonender Prozess über die Sektorengrenzen und ggf. auch über die Grenzen der Sozialgesetzbücher hinweg ermöglicht werden.

### Status quo in Deutschland

Der demografische Wandel betrifft auch die Leistungserbringer des Gesundheitswesens: Zur Aufrechterhaltung des Versorgungsniveaus von 2016 müssten bis 2035 etwa 146.000 Ärzte altersbedingt ersetzt werden [9]. Ähnliche Probleme zeigen sich auch im Nachbarland Frankreich: Hier wird die Versorgung in strukturschwachen Gegenden mit bereits pensionierten, jedoch weiterhin behandelnden Ärzten aufrechterhalten [10]. Die bisherige Versorgungsdichte in Deutschland (4,3 Ärzte bzw. 13,2 Krankenpflegekräfte/1000 Einwohner), die deutlich oberhalb des Durchschnitts der Organisation für wirtschaftliche Zusammenarbeit und Entwicklung (OECD) liegt, wird sich personell nicht erhalten lassen, weshalb der Ausbau digitaler Versorgungsangebote eine Entlastung bieten könnte [11]. Dies hat das Bundesministerium für Gesundheit in den letzten Jahren gesetzlich verankert. So sieht u. a. das Digitale-Versorgung-Gesetz (DVG) ein verpflichtendes digitales Netzwerk im Gesundheitsbereich, eine Verbesserung der Versorgungsforschung durch zentrale Sammlung und Bereitstellung von Daten der gesetzlichen Krankenversicherung (GKV) sowie die weitere Förderung von Innovationen seitens der Krankenkassen und des Innovationsfonds vor. Des Weiteren erkannte die Bundesregierung auch den Investitionsbedarf zur Verbesserung der digitalen Infrastruktur in den Krankenhäusern (Krankenhauszukunftsgesetz; KHZG) und den Pflegeeinrichtungen (Pflegepersonal-Stärkungsgesetz; PpSG). Insbesondere hier könnte das Personal durch effizientere Dokumentationsvorgänge maßgeblich entlastet werden, was wiederum der Versorgungsqualität von pflegebedürftigen Menschen zugutekäme. Neben der Optimierung einer zeitnahen Patientenversorgung können digitale Anwendungen wie die Telemedizin eine weitere Lücke schließen: In der älter werdenden Bevölkerung nehmen komplexe Krankheitsbilder zu. Die Akutgeriatrie mit ihrem um Ganzheitlichkeit und Frührehabilitation bemühten Behandlungsansatz zeigte hier bspw. 2017 mit einer durchschnittlichen Verweildauer von 15,3 Tagen die höchste Bettenauslastung (~89 %) der allgemeinen Fachabteilungen [12]. Bei weiterer Zunahme der Fallzahlen allgemein und auch im geriatrischen Bereich ist die alleinige Kompensation im stationären Sektor kaum realisierbar, was eine Ambulantisierung zwingend erforderlich macht. Hierbei wird die Telemedizin unverzichtbar sein, um eine qualitativ hochwertige ambulante Versorgung zu ermöglichen [13]. Darüber hinaus kann Telemedizin helfen, die ambulant-sensitiven bzw. Pflegeheim-sensitiven Krankenhausfälle zu reduzieren (bspw. durch ärztliche Konsultation und „Anbehandlung“ leichter Infekte oder Schmerztherapie, wenn die Hausarztpraxis nicht verfügbar ist), welche einerseits ein Gesundheitsrisiko für geriatrische Patienten (u. a. Delir, krankenhausassoziierte Infektionen) und andererseits eine Herausforderung für die akut- und notfallmedizinischen Ressourcen darstellen [14–16]. Unter Zuhilfenahme etablierter *Early Warning Scores* (z. B. NEWS2 [17]) könnten die an der Patientenversorgung unmittelbar beteiligten Akteure ein adäquates, objektivierbares Monitoring des Gesundheitszustandes vornehmen. Bei akuter Verschlechterung kann dann je nach Ausprägung die jeweils vereinbarte Maßnahme (Hausarztkontakt, Televisite, Krankenhausvorstellung) bedarfsgerecht initiiert werden.

Unverzichtbar im Wandel der medizinischen Versorgungsangebote sowie am Sektorenübergang bleiben Hausärzte mit ihrem hermeneutischen Fallverständnis. Die im Jahr 2007 eingeführte Hausarztzentrierte Versorgung (HZV) bot bereits einen vielversprechenden Ansatz (u. a. niedrigere Hospitalisierungsraten in Baden-Württemberg), die bundesweite Umsetzung verlief jedoch schleppend [18]. Um der Verantwortung als primäre Ansprechpartner in allen Belangen sowie als Spezialisten für chronische Erkrankungen und Multimorbidität nachkommen zu können, bedürfen vor allem die Hausärzte zusätzlicher digitaler Handlungsspielräume.

### Intersektorale Projekte in Deutschland

**Figure 2:**
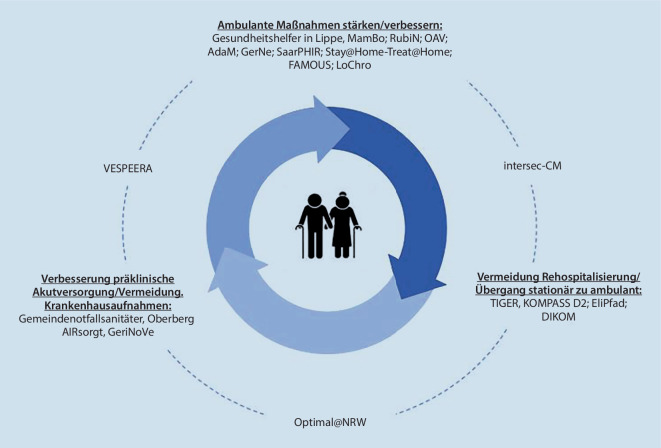

Lücken entstehen häufig am Übergang von stationärer zu ambulanter Versorgung. Da die Altersmedizin einen der höchsten Anteile der internistischen Betten in deutschen Krankenhäusern stellt [19], erwächst hieraus eine große Zahl von Patienten, die ein optimiertes Entlass- bzw. Versorgungsmanagement benötigen – die Evidenz hierzu war jedoch lange Zeit überschaubar [8]. In den letzten Jahren entstand eine Vielzahl wissenschaftlich begleiteter Projekte, die auf die verbesserte Versorgung geriatrischer Patienten am Sektorenübergang fokussierten (Abb. 2). Hierbei ist neben dem Bundesministerium für Bildung und Forschung (BMBF) der 2016 eingeführte Innovationsfonds des Gemeinsamen Bundesauschusses (G-BA) mit der gezielten Förderung von Innovationen in der GKV der größte Sponsor für die Erprobung neuer Versorgungsformen.

#### Fokus: *Transitional Care*

Eine der ersten umfassenden Studien auf diesem Gebiet war das Projekt *TIGER – Transsektorales Interventionsprogramm zur Verbesserung der geriatrischen Versorgung in Regensburg* [20]. Untersucht wurde u. a. die Anzahl der vermeidbaren Rehospitalisierungen durch die Etablierung von geschulten Fachkräften (*Pfadfindern*). Diese begleiten strukturiert den Übergang geriatrischer Patienten von stationärem Aufenthalt nach Hause und koordinieren im Folgejahr alle Fragen bezüglich gesundheitlicher und pflegerischer Versorgung (z. B. Arztkontakte, Wohnumfeldoptimierung etc.).

Bereits seit 2010 besteht in der Region Lippe die Initiative *Regionales Versorgungskonzept Geriatrie – Gesundheitshelfer in Lippe* [21]. Diese umfasst ein sektorenübergreifendes Case-Management durch Gesundheitshelfer u. a. für zu Hause lebende ältere Menschen, um deren Pflegebedürftigkeit zu verringern bzw. hinauszuzögern. Ursprünglich durch das Klinikum Lippe sowie das Ärztenetz in der Region initiiert, ist das Projekt inzwischen in eine gemeinsame Gesellschaft übergegangen und 90 niedergelassene Ärzte schreiben Patienten in das Projekt ein. Einen ähnlichen Ansatz verfolgte das Projekt *MamBo – Multimorbide Menschen in der ambulanten Betreuung: Patientenzentriertes, Bedarfsorientiertes Versorgungsmanagement* in der Region Leverkusen [22]. Mittels Monitoring- und Koordinationsassistenten (*MoniKa*) sollte das Bedarfsmanagement gesteuert, Praxispersonal entlastet und multimorbide Personen gezielt unterstützt werden. Darüber hinaus wurde eine digitale Vernetzung der ärztlichen Akteure im regionalen Gesundheitsnetz implementiert.

Auch das Projekt *RubiN – Regional ununterbrochen betreut im Netz* analysierte Struktur- und Prozessanforderungen, um aus der Arztpraxis heraus ein sektorenübergreifendes Netzwerk zur Versorgung geriatrischer Patienten in 8 Modellregionen zu realisieren [23]. Hier sollen ärztliche und nicht-ärztliche, geriatrisch versierte Fachkräfte in einer rechtssicheren Organisationsform agieren können; die Grundlage für die individuell einzuleitenden Maßnahmen der rund 3200 Teilnehmer bildet die Erhebung der individuellen Risiken durch ausgebildete Fallmanager im stationären und ambulanten Setting [23].

In 9 Landkreisen in Baden-Württemberg stand der Übergang von ambulanter zu stationärer Therapie im Fokus des Projekts *VESPEERA – Versorgungskontinuität sichern – Patientenorientiertes Einweisungs- und Entlassmanagement in Hausarztpraxen und Krankenhäusern* [24]. Durch strukturierte Informationen bei Krankenhauseinweisung sollten wichtige Aspekte für die Behandlung mitgegeben und eine proaktive Entlassplanung ermöglicht werden. Nach der Entlassung erfolgte ein Assessment in der Hausarztpraxis und darauf aufbauend die Entwicklung eines Maßnahmenkatalogs, der durch das Case-Management koordiniert wurde und negativen Outcomes entgegenwirken sollte.

Die Subgruppe geriatrischer Personen mit kognitiven Beeinträchtigungen bedarf noch mehr Aufmerksamkeit, um negativen gesundheitlichen oder sozialpflegerischen Effekten während des stationären Aufenthalts und nach Entlassung zuvorzukommen [8]. Hierzu sind in letzter Zeit zahlreiche Projekte zur Gestaltung demenzsensibler Krankenhäuser und demenzsensibler Pflege zu verzeichnen. Im Projekt *intersec-CM – Intersectoral Care Management* werden durch qualifiziertes Studienpersonal abhängig vom individuellen Assessment der Patienten Versorgungs- und Behandlungspläne für den poststationären Verlauf erstellt und die Probanden bei deren Umsetzung unterstützt [25].

Im Projekt *FAMOUS – Fallbezogene Versorgung multimorbider Patientinnen und Patienten in der Hausarztpraxis durch Advanced Practice Nurses (APN)* wird das hierzulande noch unbekannte Berufsbild der akademisch ausgebildeten Pflegenden im ambulanten Setting erprobt [26]. Patienten mit komplexen Multimorbiditäten werden individuell durch eine APN begleitet, wodurch eine Stabilisierung der Versorgung sowie eine Entlastung der Hausärzte erreicht werden soll.

Multimorbide ältere Menschen, deren Versorgungssituation und Arzneimitteltherapie stehen auch im Fokus des Projekts *LoChro – Lokales gestuftes Versorgungsmanagement bei chronisch erkrankten älteren Menschen* [27]. Mit der Implementierung einer lokal koordinierten Verbundversorgung soll eine sektorenübergreifende Verbesserung der Versorgung bewirkt werden. Hierzu werden u. a. die Inanspruchnahme und Kosten von Gesundheitsleistungen, der Funktionsstatus sowie individuelle Zufriedenheit mit der Versorgung analysiert.

#### Fokus: Arzneimitteltherapie

Die intersektorale Koordination der Arzneimitteltherapie ist ein weiteres Kernthema – nicht zuletzt wegen des geriatrischen Syndroms der Polypharmazie und den damit verbundenen Gesundheitsrisiken.

Im Projekt *OAV – Optimierte Arzneimittel-Versorgung pflegebedürftiger Patienten* soll ein klinisches Risikomanagementsystem der Medikation aufgebaut werden, um arzneimittelassoziierte Erkrankungen bzw. unerwünschte Arzneimittelereignisse zu reduzieren. 96 interdisziplinäre geriatrische Teams in Modellregionen in 4 Bundesländern stimmen darauf basierend die weitere Behandlung der ca. 4800 pflegebedürften Patienten ab [28].

Mit dem Projekt *AdAM – Anwendung für digital unterstütztes Arzneimitteltherapie-Management* wurde Hausärzten ein digitales Tool zur Verfügung gestellt, welches eine strukturierte, vollständige Übersicht über alle verordneten Medikamente sowie Heil- und Hilfsmittel bietet. Darüber hinaus werden die teilnehmenden Ärzte automatisch über Krankenhausaufnahmen ihrer Patienten informiert. Algorithmen, bspw. Risikowarnungen, sollen eine bedarfsgerechtere Verordnung ermöglichen, was zu einer Reduktion von arzneimittelbezogener Mortalität führen kann [29].

#### Fokus: Versorgung mit Telemedizin

Wie bereits oben dargestellt, wird die Telemedizin zunehmend utilisiert werden müssen, um kritische Versorgungslücken durch limitierte personelle Ressourcen zu schließen. Im Projekt *Oberberg_FAIRsorgt* (Modellregion Oberbergischer Kreis) werden pflegebedürftige Menschen und deren Angehörige sektorenübergreifend medizinisch und pflegerisch mittels telemedizinischer Angebote unterstützt [30].

Das Projekt *GerNe – Geriatrisches Netzwerk* vernetzt stationäre und ambulante Versorger von geriatrischen Patienten in Rheinland-Pfalz. Basierend auf einer elektronischen Patientenakte findet ein regelmäßiger Austausch zwischen Hausärzten, Geriatern und Apothekern statt und im Bedarfsfall steht ein geriatrischer Konsildienst zur Verfügung. Hierdurch soll die Anzahl der (Re)Hospitalisierungen reduziert werden [31].

Regelmäßige, multidisziplinäre telemedizinische Konsile bilden auch das Kernelement des Projekts *KOMPASS D2 – Komplikations-Management und Prävention im Ambulanten und Stationären Sektor – Demenz & Delir* [32]. Gezielte Präventionsmaßnahmen sowie strukturiertes Delirmanagement sollen helfen, die Delirrate bei der Versorgung geriatrischer Patienten zu reduzieren. Darüber hinaus ist jeweils eine geschulte Pflegekraft den 2400 Patienten fest zugeordnet und begleitet diese während und nach dem stationären Aufenthalt.

Im Saarland zielt das Projekt *SaarPHIR – Saarländische Pflegeheimversorgung Integriert Regelhaft* auf die verbesserte Zusammenarbeit von Ärzten und Pflegekräften, indem fest zugeordnete regionale Versorgerteams gebildet werden, die sich regelmäßig über die Bewohner austauschen (inklusive Arzneimittelüberprüfung; [33]). Die teilnehmenden Ärzte können so Ressourcen bündeln und eine verbesserte Erreichbarkeit für die Pflegeheime realisieren.

In Nordrhein-Westfalen ist mit *EliPfad – Personalisierter, interdisziplinärer Patientenpfad zur sektorenübergreifenden Versorgung multimorbider Patienten mit telemedizinischem Monitoring* an 6 Kliniken ein Projekt gestartet, welches bei multimorbiden Patienten nach einem Klinikaufenthalt mittels digitaler Anwendungen eine Verringerung der Rehospitalisierungsrate sowie einen längeren, selbstständigen Verbleib in der Häuslichkeit zum Ziel hat [34].

Der Verbleib in der gewohnten Umgebung ist insbesondere für Pflegeheimbewohner ein wichtiges Element, um negativen gesundheitlichen Effekten, wie bspw. Delir, vorzubeugen. Bei den häufig multimorbiden Patienten ist jedoch nicht selten apparative Diagnostik notwendig, um potenziell instabile Komorbiditäten zu evaluieren und ggf. angepasste Behandlungsstrategien einzuleiten. Das Projekt *DIKOM – Diagnostik und Konsil im Pflegeheim mittels Mobiler Geriatrie-Unit* bietet hierfür eine Lösung, um apparative Untersuchungen vor Ort durchzuführen und wird von Fachärzten sowie medizinischen Technologen für Radiologie (MTR) besetzt [35]. Die erhobenen Befunde werden an die entsprechenden Mitbehandler übermittelt und per telemedizinischem Konsil beraten.

#### Fokus: Akutmedizinische Versorgung und angemessene Versorgungsstufe

Pflegebedürftige Menschen sind durch die häufig assoziierte Multimorbidität regelmäßig von akutmedizinischen Ereignissen betroffen. Nicht selten ist die Wahl der angemessenen Versorgungsstufe eine Herausforderung für die Betroffenen bzw. Pflegeverantwortlichen. Hier setzen mehrere Projekte an. In Berlin wird mit *Stay@Home-Treat@Home* ein telemedizinisches Netzwerk aufgebaut, in dem rund um die Uhr die Versorgung ambulant pflegebedürftiger Menschen durch die entsprechenden Leistungserbringer mit abgestuften Maßnahmen sichergestellt werden soll [36]. Je nach Dringlichkeit des Meldebildes wird die geeignete Versorgungsstufe zur Unterstützung alarmiert.

Im Projekt *Optimal@NRW – Optimierte Akutversorgung geriatrischer Patienten durch ein intersektorales telemedizinisches Kooperationsnetzwerk* im Raum Aachen soll durch den Aufbau eines telemedizinischen Netzwerks die bedarfsgerechte medizinische Akutversorgung stationär pflegebedürftiger Patienten verbessert werden [16]. Das Projekt versucht durch Einbeziehung der Pflegekräfte als Anwender die Akzeptanz zu steigern und einen Bogen zwischen Pflege, Patientenwillen und medizinischer Behandlung zu schlagen. Ambulant-sensitive Krankenhausfälle sollen reduziert und Krankheitsbilder mithilfe der sektorenübergreifenden Position der nicht-ärztlichen Praxisassistenz mit Zusatzaufgaben (NäPa(Z)) anbehandelt werden, die rund um die Uhr ärztlich delegierbare Maßnahmen in den Pflegeeinrichtungen vornehmen kann.

In Niedersachsen wird die Verbesserung der präklinischen Akutversorgung bei nicht lebensbedrohlichen Notfällen durch den Einsatz eines *Gemeindenotfallsanitäters* erprobt [37]. Dieser kann auf ein Netzwerk ambulanter Strukturen zurückgreifen, um eine Behandlung zu initiieren bzw. selbst vor Ort durchzuführen, was insbesondere für geriatrische Patienten unnötige Krankenhaustransporte reduzieren kann.

Für die besondere Situation einer akuten sozialpflegerischen Krise ohne unmittelbaren medizinischen Notfall hat das Projekt *GeriNoVe – Geriatrisches Notfall-Versorgungszentrum* in Baden-Württemberg ein Zentrum etabliert, in dem kurzfristig eine Versorgung durch ein interdisziplinäres Team sichergestellt werden kann [38]. Durch die sektorenübergreifende Vernetzung von ambulanten und stationären Leistungserbringern sowie kommunalen Diensten soll eine zeitnahe Überführung in die geeignete Versorgungsform (im besten Fall zurück in das häusliche Umfeld) realisiert und somit gefährliche Versorgungslücken vermieden werden.

### Von der Wissenschaft in die Regelversorgung – Hürden und Chancen

Die Vielzahl der gut durchdachten Konzepte wurde bisher noch nicht in die Regelversorgung übernommen. Unter anderem wird das starre und zergliederte System unseres Gesundheitswesens als Grund angegeben [21]. Kernelement vieler Projekte ist die Bildung von Netzwerken zum verbesserten Informationsaustausch zwischen Sektoren und versorgenden Institutionen, deren Effekt sich jedoch nur erschwert erfassen lässt [39]. Allerdings beansprucht die Implementierung im Rahmen der Projekte viele Ressourcen bei der Überwindung technischer oder datenschutzrechtlicher Hindernisse. Doch gerade diese Vernetzung zeigte im Einzelnen positive Effekte: In vielen Projekten wurde beschrieben, dass die Kooperation von Ärzten, anderen Fachkräften und Kliniken einer Region zur verbesserten Versorgung von Patienten geführt hat [21]. Dies kann jedoch unter Umständen bedeuten, dass Erkrankungen besser erkannt und stationäre Behandlungen eingeleitet wurden, was wiederum für die Kostenträger keine Ersparnis darstellt und somit nicht als erstrebenswerter Endpunkt aus gesundheitsökonomischer Perspektive gewertet wird. Die Bewertung der Kosteneffektivität bedarf möglicherweise einer komplexeren Betrachtung, denn eine kurzfristige Mehrausgabe induziert ggf. eine Kostenreduktion im späteren Krankheitsverlauf durch die frühzeitige Intervention – dieser longitudinale Effekt kann jedoch mit den o. g. Projekten nicht abgebildet werden. Auch bezüglich der Finanzierung der Leistungserbringenden bestehen Unsicherheiten. Eine systematische Untersuchung über den Grad der Translation von Telemedizinprojekten beschreibt, dass wissenschaftlich geleitete Forschungsprojekte (z. B. durch Unikliniken) durch die sachgrundbezogenen Projektmittel und Arbeitsverträge keine finanziellen und personellen Ressourcen haben, um ihre Konzepte nach Projektende im Versorgungsalltag zu implementieren [40]. Hierdurch enden Ansätze mit Ablauf der Drittmittelfinanzierung und die im Laufe der Projektzeit erworbenen Kompetenzen gehen verloren.

Darüber hinaus ist die wissenschaftliche Begleitung der Projekte bislang leider nicht vollumfänglich in Publikationen abgebildet, woraus für die forschende Öffentlichkeit wichtige ableitbare Erkenntnisse teilweise verborgen bleiben. Bei dieser Einschätzung muss allerdings berücksichtigt werden, dass eine deutliche Verzögerung zwischen Abschluss der Studien und Erstellung des Abschlussberichts unvermeidbar ist und eine vorzeitige Veröffentlichung von (Teil‑)Daten untersagt oder unerwünscht ist, um eine unabhängige Gesamtevaluation zu gewährleisten. Insofern sind für die sich aktuell in Abschluss befindenden Projekte hoffentlich noch gewinnbringende Veröffentlichungen in der näheren Zukunft zu erwarten. Tab. 1 zeigt eine Übersicht der aktuellen Studien und Empfehlungen zu bereits abgeschlossenen Projekten aus dem ersten Förderzeitraum des Innovationsfonds. Trotz Reduktion der zur Verfügung stehenden Mittel ist die Nachfrage zur Förderung ungebrochen, was den hohen Innovationsbedarf im Gesundheitswesen unterstreicht [41]. Mit der zu erwartenden Verstetigung dieser Förderstruktur sowie den Erkenntnissen und Erfahrungen aus bisherigen Projekten ohne Empfehlung zur Überführung in die Regelversorgung bleibt zu hoffen, dass neue Projekte entsprechende Hürden erfolgreich überwinden, um die Gesundheitsversorgung im Spannungsfeld der zahlreichen Herausforderungen weiter zu verbessern.

**Table 1:** 

Projektname	Status	Förderer	Fördersumme	Empfehlung	Kommentar zur Empfehlung
*TIGER*	Abgeschlossen	G‑BA Innovationsfonds	3,7 Mio. €	–	Kein positiver Effekt in der Wirksamkeitsevaluation sowie der Kosteneffektivität nachweisbar bei kleiner Stichprobe und unvollständigen Daten; positive Bewertung der Intervention bei allen Projektteilnehmenden
*Gesundheitshelfer Lippe*	Abgeschlossen/alternative Anschlussfinanzierung	Land NRW/Klinikum Lippe/Ärztenetz Lippe	n/a	n/a	Seit Implementierung stetige Erweiterung der Kompetenzen und Zielgruppe (Entlassmanagement, seit 2016 komplex erkrankte Menschen aller Altersstufen, seit 2014 Pflegeheimbewohner)
*MamBo*	Abgeschlossen	G‑BA Innovationsfonds	3,4 Mio. €	–	Implementierung in den Praxen problematisch, eingeschränkte Inanspruchnahmemöglichkeit der Intervention; methodische Schwächen bei der Wirksamkeitsevaluation, positive Bewertung der Patienten insbesondere während der Pandemie
*RubiN*	Abschlussbericht wird erstellt	G‑BA Innovationsfonds	8,1 Mio. €	n/a	Neben Wirksamkeitsanalyse auch Analyse der Rechtsgrundlagen für die neue Versorgungsform
*VESPEERA*	Abgeschlossen	G‑BA Innovationsfonds	3,8 Mio. €	–	Keine signifikante Reduktion der Rehospitalisierung, Implementierung nur teilweise gelungen, somit Verzerrung der Ergebnisse; methodische Schwächen der Effektevaluation, kleine Stichprobe
*intersec-CM*	Abgeschlossen	BMBF	1,7 Mio. €	n/a	Deskriptive Analyse der Baseline-Daten von 402 Probanden zeigt hohen Anteil älterer Patienten im Krankenhaus mit kognitiven Beeinträchtigungen und entsprechendem Hilfebedarf, der zum Zeitpunkt der Hospitalisierung nicht bedarfsgerecht adressiert war [42]
*OAV*	Abgeschlossen	G‑BA Innovationsfonds	6,5 Mio. €	(–)	Erfolgreiche Implementierung mit begleitender Qualitätssicherung, positive Tendenzen der Evaluationsergebnisse, methodische Schwächen bezüglich der Datenbasis der Evaluation, Weitergabe der Erkenntnisse u. a. an Hausärzteverband e. V.
*AdAM*	Abgeschlossen	G‑BA Innovationsfonds	16,3 Mio. €	+	Empfehlung zur Überführung von Ansätzen in die Regelversorgung bei nachgewiesener Reduktion der Gesamtmortalität durch Intervention (jedoch mglw. Selektionsbias), positive Bewertung der Studienteilnehmenden, insbesondere Ärzte berichten höhere Sensibilisierung bzgl. Multimedikation; Erstellung S2k-Living Guideline Arzneimitteltherapie bei Multimorbidität
*LoChro*	Abgeschlossen	BMBF	1,5 Mio. €	n/a	Kein statistischer Effekt hinsichtlich primärer und sekundärer Endpunkte; zukünftige Ansätze sollten proaktiveres Case-Management oder frühzeitigere Intervention hinsichtlich Care-Management berücksichtigen
*Oberberg FAIRsorgt*	Laufend	G‑BA Innovationsfonds	11,2 Mio. €	n/a	n/a
*GerNe*	Abschlussbericht wird erstellt	G‑BA Innovationsfonds	3,8 Mio. €	n/a	n/a
*KOMPASS D2*	Laufend	G‑BA Innovationsfonds	5,6 Mio. €	n/a	n/a
*SaarPHIR*	Abschlussbericht wird erstellt	G‑BA Innovationsfonds	5,5 Mio. €	n/a	n/a
*EliPfad*	Laufend	G‑BA Innovationsfonds	12,7 Mio. €	n/a	n/a
*DIKOM*	Laufend	G‑BA Innovationsfonds	9,8 Mio. €	n/a	n/a
*Stay@Home Treat@Home*	Laufend	G‑BA Innovationsfonds	9 Mio. €	n/a	n/a
*Optimal@NRW*	Abschlussbericht wird erstellt	G‑BA Innovationsfonds	14,9 Mio. €	n/a	n/a
*ILEG*	Abschlussbericht wird erstellt	G‑BA Innovationsfonds	1,1 Mio. €	n/a	n/a
*GeriNoVe*	Abschlussbericht wird erstellt	G‑BA Innovationsfonds	4,5 Mio. €	n/a	n/a
*FAMOUS*	Laufend	G‑BA Innovationsfonds	4,2 Mio. €	n/a	n/a

*+* Empfehlung zur Weiterführung/Translation in die Regelversorgung, *–* keine Empfehlung zur Weiterführung, *(–)* nur eingeschränkt empfohlen, *n/a* noch keine Angaben hierzu vorhanden, *G‑BA* Gemeinsamer Bundesausschuss, *BMBF* Bundesministerium für Bildung und Forschung

### Ansätze und Projekte international: Gibt es andere Vorgehensweisen?^1^

Im Hinblick auf unsere mosaikartige Versorgungslandschaft lohnt sich ein Blick über die (kontinentalen) Grenzen hinweg, um Inspiration für mögliche Lösungen unseres Implementierungsproblems zu erhalten.

Am Beispiel Japans lassen sich die für Deutschland und Europa noch bevorstehenden Entwicklungen gut verdeutlichen: Japan besitzt im internationalen Vergleich eine der ältesten Bevölkerungen^2^ [43]. Bereits im Jahr 2013 hat die japanische Regierung eine Reform des Gesundheitswesens erlassen. Die Reform verteilt die Finanzierung des medizinischen Systems hinsichtlich der Belastung für einzelne Gruppen neu und setzt auf die Entwicklung neuer Behandlungsstrategien zur besseren Therapie chronischer Krankheiten [44]. Durch die neue Finanzierung wurden langfristig neue Ressourcen geschaffen, um die medizinische Ausbildung in den Bereichen Geriatrie und Gerontologie voranzutreiben [45]. An vielen Ausbildungsstätten des Landes wurden feste Curricula für diese Fachbereiche integriert und die Anzahl der auf Altersmedizin spezialisierten Fachkräfte konnte gesteigert werden [44]. Es wird deutlich, dass Japan die zukünftigen Effekte der Reform priorisiert und auf langfristige statt auf kurzfristige Kostenreduktion setzt.

Eine Initiative der niederländischen Regierung förderte ab 2008 wissenschaftliche Arbeiten zur besseren Versorgung der alternden Bevölkerung im Rahmen der medizinischen Grundversorgung. Das Project *ACT – Adult: Care in Transition* etablierte ein Modell für die Versorgung chronisch Kranker in den Alltag geriatrischer Patienten, z. B. mit regelmäßigem geriatrischen Assessment und hierauf aufbauenden personalisierten Therapieplänen [46]. *Prevention of Care (PoC)* der Universität Maastricht versuchte über einen ähnlichen Ansatz mit multimodalem Assessment in der Häuslichkeit den Grad der Behinderung der Zielgruppe positiv zu beeinflussen sowie die Selbstständigkeit zu erhalten [47]. Beide Projekte erwiesen sich jedoch nicht als kosteneffektiv [48]. Diese Ergebnisse betonen (ähnlich wie in Deutschland) die Schwierigkeit, Interventionseffekte in dieser speziellen Patientengruppe zu erfassen.

Einen Lichtblick hinsichtlich Intersektoralität bietet Skandinavien: So gibt es bspw. in Schweden und Norwegen zentrale Stellen (teilweise Aufgabe der Hausärzte), die eine Vermittlung von Arztkontakten, Krankenhausvorstellung oder Pflegeleistungen vornehmen [49]. Hierdurch ergeben sich weniger Hürden in Bezug auf Intersektoralität, denn diese ist bereits das Grundkonstrukt des Systems. Somit können Kosten reduziert und Patientenströme besser gesteuert werden.

## Schlussfolgerung: Beginn bei vernetzter Forschung

Anhand der aufgeführten Beispiele zeigt sich, dass deutliche Anstrengungen zur Überwindung intersektoraler Grenzen für ältere Personen unternommen und digitale Lösungen angestrebt werden. Kurzfristig nachzuweisende Interventionseffekte oder stetiger Kostendruck auf Projekte in den Implementierungsjahren erschweren jedoch die Verknüpfung der Sektoren und stehen der verbesserten Versorgung geriatrischer Patienten im Weg, sodass Ideen, Fortschritte sowie auch potenzielle Hürden im kleinen Rahmen verbleiben.

Die Mehrzahl der beschriebenen Projekte in Deutschland wird bzw. wurde aus Mitteln des Innovationsfonds des G‑BA gefördert. Teilweise gibt es eine deutliche Überschneidung bezüglich Patientenauswahl, Interventionsmaßnahmen und evaluierter Outcomes. Bereits abgeschlossene Projekte haben – mit Ausnahme von *AdAM* – keine grundsätzliche Empfehlung zur Verstetigung erhalten. Zum Teil werden die Maßnahmen auf Kosten der Kliniken oder im Rahmen von Selektivverträgen regional fortgeführt, was jedoch partiell eine ethisch fragwürdige Ungleichbehandlung von Patienten bedeutet [50]. Um zügig eine möglichst flächendeckende Optimierung zu erreichen, erscheint eine niedrigschwellige Freigabe in die Regelversorgung und die stärkere Vernetzung der wissenschaftlichen Akteure auf diesem Gebiet erstrebenswert. So könnten sich Synergieeffekte aus Forschung, Projektdurchführung und Evaluation besser entfalten und die Evidenzgenerierung als schlagkräftige Argumente für die Verstetigung von Versorgungsformen vorangetrieben werden.

## Fazit

Der aktuelle Trend zur intersektoralen Zusammenarbeit im Hinblick auf die geriatrische Patientenklientel wird auch zukünftig ein essenzieller Aspekt sein, um unser Gesundheitssystem auf die bevorstehenden Veränderungen anzupassen. Auch der wachsende Telemedizinanteil wird für die Kostenreduktion und Entlastung der abnehmenden Anzahl von Leistungserbringern im Gesundheitssektor unverzichtbar werden. Doch um das Ziel der intersektoralen Versorgung zu erreichen, müssen alle Akteure in Gesundheitsversorgung und Forschung organisatorische und gedankliche Hürden überwinden. Wenn wie im internationalen Vergleich hierzulande die Intersektoralität besser im Gesamtsystem verankert wird und die kostenbezogenen Effekte langfristiger betrachtet werden, dann gibt es bereits heute zahlreiche Modelle, die unsere Versorgungslandschaft für geriatrische Personen nachhaltig verbessern können.

## Footnotes

1 Die angebrachten Beispiele sind aufgrund grundlegender Unterschiede der Gesundheitssysteme nur eingeschränkt auf das deutsche System übertragbar und sollen daher nur als Impuls für innovative Gedanken verstanden werden.

2 2020 betrug die durchschnittliche Lebenserwartung für Frauen 87,7 Jahre.
